# Who's My Daddy? Considerations for the influence of sexual selection on multiple paternity in elasmobranch mating systems

**DOI:** 10.1002/ece3.3086

**Published:** 2017-06-15

**Authors:** Kady Lyons, Chris L. Chabot, Christopher G. Mull, Corinne N. Paterson Holder, Christopher G. Lowe

**Affiliations:** ^1^ California State University, Long Beach Long Beach CA USA; ^2^ California State University, Northridge Northridge CA USA; ^3^ Simon Fraser University Burnaby Canada

**Keywords:** competition, mating systems, reproductive strategies, sexual selection

## Abstract

Polyandry resulting in multiply‐sired litters has been documented in the majority of elasmobranch species examined to date. Although commonly observed, reasons for this mating system remain relatively obscure, especially in batoids. The round stingray (*Urobatis halleri*) is an abundant, well‐studied elasmobranch distributed throughout the northeastern Pacific that we used to explore hypotheses regarding multiple paternity in elasmobranchs. Twenty mid‐ to late‐term pregnant females were sampled off the coast of southern California and their litters analyzed for the occurrence of multiple paternity using five nuclear microsatellite loci. In addition, embryo sizes and their position within the female reproductive system (i.e., right or left uterus) were recorded and used to make inferences for patterns of ovulation. Multiple paternity was observed in 90% of litters and male reproductive success within litters was relatively even among sires. High variability in testes mass was observed suggesting that sperm competition is high in this species, although male reproductive success per litter appeared to be relatively even. Using embryo size as a proxy for fertilization, females were found to exhibit a variety of ovulation patterns that could function to limit a male's access to eggs and possibly promote high rates of multiple paternity. Our study highlights that elasmobranch mating systems may be more varied and complex than presumed and further investigation is warranted.

## 1 INTRODUCTION

Multiply‐sired litters can arise when several males have access to a receptive female during the reproductive season. However, studies over the past few decades have demonstrated that simple copulation does not necessarily assure sireship by individual males (Andersson and Simmons 2006). When males compete with other males for access to females, and by extension their eggs, this can lead to the development of various strategies for males to increase their reproductive success. For instance, sperm from different males can compete with each other for access to eggs within a female's reproductive system long after copulation has occurred (Parker 1970, Simmons 2001). This scenario represents a form of intrasexual competition, which leads to differential access to females by certain males over others. On the other hand, females can also enact strategies to bias which males are allowed to fertilize her eggs, known generally as cryptic female choice (Birkhead, 1998). In this form of intersexual selection, females can favor and/or discriminate among males preferentially through a variety of mechanisms (i.e., behaviorally, physiologically, or morphologically). Sexual selection, in any of its forms, can have consequences for litter paternity as any factor that promotes or inhibits sperm's access to eggs will influence sireship.

In marine and freshwater systems, multiple paternity has been documented across fish species utilizing various forms of reproductive strategies (Coleman & Jones, 2011). In species utilizing external fertilization, rates of multiple paternity would be expected to be high, due to the lack of control females have over sperm access to eggs in an open environment. However, multiple paternity appears to also occur at a high rate in fishes where fertilization is internal. For example, elasmobranchs (sharks and rays) are a group of cartilaginous fishes that use internal fertilization where multiple paternity has been observed in the majority of species studied to date (see Byrne & Avise, 2012 and Fitzpatrick, Kempster, Daly‐Engel, Collin, & Evans, 2012 for review). Due to aggressive male mating behaviors and the physical rigors of copulation (Pratt & Carrier, 2001), the physical cost of mating is assumed to be high for female elasmobranchs. Therefore, one hypothesis for the high rates of multiple paternity in elasmobranchs has been attributed to “convenience” (DiBattista, Feldheim, Gruber, & Hendry, 2008; Feldheim, Gruber, & Ashley, 2004), where the cost of female resistance to copulation outweighs the cost of multiple matings and leads to multiple males siring individual litters.

In systems exhibiting multiple paternity, male reproductive success will vary depending on an individual male's access to females and the opportunity to fertilize eggs. In species with skewed reproductive success (i.e., uneven access among males to females), high sperm competition results in heavier, and more variable, testes mass among males compared to other populations or congeners where reproductive success is more equal (Harcourt, Harvey, Larson, & Short, 1981; Parker, Ball, Stockley, & Gage, 1997). The positive relationship between testes weight and incidence of multiple paternity in elasmobranchs suggests that sperm competition may be an important factor for this phenomenon (Fitzpatrick et al., 2012). However, this has not formally been examined with respect to male sireship potential (i.e., the percent of a litter an individual male sires).

In a system where a given male's only contribution to the reproductive process is the production of sperm, male elasmobranchs would be expected to seek multiple mating opportunities to increase their fitness. On the other hand, female elasmobranchs would be expected to limit the number of males they mate with due to the physical rigors of copulation and the amount of energy that viviparous females must expend to carry offspring to term. As elasmobranchs do not provide parental care postpartum, benefits (or costs) gained by females from multiple matings would be assumed to be genetic in nature. While females may not be able to rebuff all male advances during the mating season, they could potentially utilize other sexual selection strategies to exert some control over the ability of certain males to fertilize a given litter. One potential strategy may be the alteration of sperm access to eggs, a form of cryptic female choice. Some elasmobranchs are documented to have extended ovulation periods that last weeks (Castro, 2000) or extended mating periods that do not coincide with ovulation (Kajiura, Sebastian, & Tricas, 2000). As a result, females may be able to affect the relative contribution of any given male to the overall composition of a litter and increase the genetic diversity of a litter by way of a variety of mechanisms (i.e., differences in ovulation timing, sperm storage, etc.). Despite the existence of this physiological mechanism, influences on multiple paternity from the female perspective generally go unconsidered.

While multiple paternity within litters appears to be the norm rather than the exception in elasmobranchs (Byrne & Avise, 2012; Fitzpatrick et al., 2012), very little attention has been focused on batoids, despite the varied habitats and modes of reproductive investment utilized by this group. In addition, the results of the few studies that have examined multiple paternity in batoids may have been somewhat confounded as they all have been influenced by the use of captive individuals (Chevolot, Ellis, Rijnsdorp, Stam, & Olsen, 2007; Janse et al. 2013). Studies based on captive animals would be expected to artificially alter mating opportunities resulting in underestimation of multiple paternity and reproductive success among males due to a limited number of individuals within the breeding captive population. Based on this, it is imperative that an investigation of multiple paternity be conducted within a wild population of batoids to provide a more realistic estimate of the frequency of multiple paternity within the group.

Considering that female reproductive investment can be altered depending on paternal–maternal genome conflicts (Zeh & Zeh, 1996), multiple paternity in stingrays is worthy of consideration as female rays utilize a unique form of supplemental nutrition (i.e., the secretion of lipid histotroph, or uterine milk; Wourms & Bodine, 1983). In histotrophy, a female's investment occurs both prior to and after fertilization with siblings potentially competing for resources. Unlike placental viviparity seen in some shark species, where females may influence the nutrients provided to individual offspring postmating, and oviparity, where females cannot regulate investment postmating, histotrophic viviparity represents a high level of investment in which females can potentially regulate supplemental nutrition postfertilization but not to the level of individual offspring. Studying this unexamined mode of reproductive investment (i.e., histotrophy) may provide new perspectives into multiple paternity in other elasmobranch species.

Round stingrays (*Urobatis halleri*) are a locally available and very abundant elasmobranch in southern California waters with a large population comprised of mobile individuals (Lowe et al., 2007; Plank, Lowe, Feldheim, Wilson, & Brusslan, 2010; Vaudo & Lowe, 2006). As the reproductive biology of this species has been fairly well investigated, key details on evidence of sperm storage (Babel, 1967), variation in testes mass during reproductive and quiescent periods (Lyons, 2013; Mull, Lowe, & Young, 2010), and number of offspring within litters and their gestational development (Lyons, 2013) are currently known. Furthermore, female round stingrays have been observed to mate with multiple males in the field (Nordell, 1994; T. Tricas, pers comm) and based on these observations we expect that multiple paternity will be observed in this species. With the availability of the above data, we feel that the round stingray will provide insight into the mating strategies of wild batoids and enhance our overall understanding of elasmobranch reproduction.

## 2 METHODS

### 2.1 Sample collection

Pregnant females (*n* = 20) were collected from two local estuaries in southern California (Colorado Lagoon and Seal Beach National Wildlife Refuge) from 2010 to 2011. Round stingrays were collected by beach seine and transferred to the Shark Lab at California State University, Long Beach, where they were subsequently euthanized. All collecting, handling, and euthanasia procedures were in accordance to CSULB animal care committee approved guidelines (IACUC approved protocol #273). Embryos were removed from the uteri, and their placement (e.g., right or left side) was recorded as well as their weight and disk width. Fin clips from embryos and muscle tissue from mothers were taken and placed in 95% ethanol and were stored at 4°C until DNA extraction.

### 2.2 DNA extraction and amplification

DNA extractions were carried out using the DNEasy Blood and Tissue Kit (Qiagen, USA) following the manufacturer's protocol. Five microsatellite loci (Uha 20, Uha 36, Uha 61, Uha 111, and Uha 115) developed for round stingrays (Plank et al., 2010) were amplified in 10 μl PCRs in an Eppendorf^®^ Mastercycler^®^ Pro vapo.protect™ thermal cycler. Each locus was amplified using a three‐oligonucleotide primer polymerase chain reaction (PCR) system consisting of a fluorescent label (6FAM or 5HEX) with an oligonucleotide tag (5‐Label‐CGAGTTTTCCCAGTCACGAC‐3), a forward primer with a long tail (5‐CGAGTTTTCCCAGTCACGAC‐3) and a reverse primer with a pig‐tail (5′‐GTTTCTT‐3′; Brownstein, Carpten, & Smith, 1996; Schuelke, 2000). Each PCR consisted of 1.8 μl of nanopure water, 2 μl of reverse oligonucleotide primer diluted to 10 μmol/L, 0.15 μl of forward oligonucleotide primer diluted to 10 μmol/L, 0.05 μl of fluorescent oligonucleotide primer diluted to 10 μmol/L, 5 μl of Type‐It Microsatellite PCR kit (Qiagen, USA), and 1 μl of template DNA with an initial denaturing step of 95°C for 5 min, followed by seven cycles of denaturing at 95°C for 40 s, with annealing temperature decreasing by 1°C for each cycle from 61°C to 55°C for 45 s, elongation at 72°C for 45 s, followed by 28 cycles of 95°C for 40 s, 55°C for 45 s, and 72°C for 45 s with a final elongation step of 72°C for 5 min. All PCR products were electrophoresed on an Applied Biosystems (ABI) 3130*xl* DNA Analyzer at California State University, Northridge, Sequencing Core. Allele sizes were determined using GeneScan™ 500 LIZ^®^ (ABI) as an internal size standard and were visualized in GENEMARKER (Softgenetics). Approximately 30% of the samples were re‐amplified to check for consistency across PCR amplifications and loci. Missing data are known to affect parentage estimates and are not tolerated by the majority of software packages used to perform these analyses. Unfortunately, the DNA quality of some pups within litters (generally 1–2) was poor and prevented all littermates from being genotyped. While we were still able to detect the presence or absence of multiple paternity with incomplete litters, we addressed paternal skew patterns in samples with a full complement of littermates genotyped (*n* = 8; litter size range 4–6) and with only one pup missing (*n* = 9; litter size range analyzed 3–6).

### 2.3 Genetic analyses

Prior to analyses, pup genotypes were compared to their respective mothers with the expectation that pups share at least one allele per locus with their mothers. Allele frequencies for all genetic analyses were based on the Seal Beach reference population (*n* = 134 samples; Plank et al., 2010). Loci Uha 20, Uha 36, Uha 61, Uha 111, and Uha 115 in the reference population from Plank et al. (2010) were in Hardy–Weinberg equilibrium, had between 10 and 37 alleles with an average of 28.6, and expected heterozygosities of 0.77, 0.87, 0.91, 0.87, and 0.89, respectively, with an average expected heterozygosity of 0.86. Alleles observed in mothers and/or pups but not in the reference population were set to a frequency of 0.01, and all other frequencies were adjusted accordingly. As the polymorphism of microsatellite loci, the number of putative fathers and their reproductive success, and the size of litters can affect the probability of detecting and quantifying multiply paternity within a litter, simulations were run in PrDM (Neff & Pitcher, 2002) to determine the power of the microsatellite markers used in this study to detect multiple paternity in litters of round stingrays. Several simulations were run with varying parameters including number of sires, reproductive skew of putative fathers (i.e., the number of different sires responsible for a single litter), and litter size. Studies of polyandry in sharks have detected a range of one to seven sires per brood (Boomer et al., 2013; Byrne & Avise, 2012; Chabot & Haggin, 2014; Chapman et al., 2013; Heist, Carrier, Pratt, & Pratt, 2011), so this study assumed a conservative range of two to five sires. Each simulation was run with litter sizes ranging between 3 and 6 that corresponded to the minimum and maximum size of observed litters.

GERUD 2.0 (Jones, 2005) was used to generate paternal genotypes and to detect the minimum number of sires per litter under an exhaustive search. In addition to GERUD 2.0, COLONY2 (Jones & Wang, 2010; Wang, 2004, 2010) was used to reconstruct putative paternal genotypes, assign paternity and sibship based on multilocus microsatellite genotypes, and to determine the maximum number of sires per litter. In order to perform these analyses, COLONY2 uses a maximum likelihood framework to assign individuals to clusters based on parent–offspring relationships, full siblings, and half siblings. As a result of this approach, clusters of full siblings are composed of all pups that share both parents and clusters of half siblings are composed of pups in which only one parent is shared. Based on these assignments, multiple paternity and reproductive skew can be inferred from the number of estimated fathers contributing to a given litter and the relative contribution of each father to a given litter. Analyses in COLONY2 were run three times to determine the consistency of the analyses with allelic dropout set to 0 and the error rate set to 0.02. As COLONY2 has been shown to overestimate the putative number of sires for a litter (Sefc & Koblmüller, 2009), comparing the minimum number of sires obtained from GERUD 2.0 with the results of COLONY2 is considered to be a test of congruence between the methodologies. High congruence between the methods is expected to provide the most likely number of sires necessary to explain paternity within an array and a lack of congruence is expected to demonstrate a range of sires with the true number of sires lying somewhere in between.

### 2.4 Morphometric analysis

As previous studies have documented that sperm competition and reproductive skewness relate to testes mass and variation, we wanted to compare the differences in variation in male testes mass between the quiescent (April–June), recrudescent (July–October), and degenerate (November–March) phases of male reproductive physiology (Mull, Lowe, & Young, 2008) as a proxy for measuring the degree of sperm competition potentially occurring. During the quiescent phase, no spermatogenesis is occurring and measures of gonadosomatic index (GSI) are at their lowest, whereas the recrudescent phase is marked by the initiation of spermatogenesis to produce secondary spermatocytes and an increase in GSI, which peaks in October. The recrudescent phase is followed by the degenerate phase and results in the breakdown of spermatagonia and decreasing GSI. Quiescent data were obtained from Lyons (2013) and Mull et al. (2008) and recrudescent and degenerate phase data from Mull et al. (2008) and Franz (2014). Males from all of these studies were sampled at the same location (i.e., Seal Beach) using the same capture methods (i.e., beach seine) and were assumed to be part of the same population based on the results of Plank et al. (2010). As male reproductive anatomy undergoes structural changes while in the same phase, we grouped male morphometric data by month for comparisons. To compare the variation in testes mass and GSI, we first performed linear regressions of either testes mass or GSI by male disk width (i.e., size) and testes mass and inner clasper length and obtained the residuals for each month. As a result, we were able to take into account variation that originates from testes mass and GSI as it scales with male disk width (i.e., size and, consequently age). The absolute value of the residuals (natural log‐transformed for normality where appropriate) for each month by metric (i.e., testes mass by disk width, testes mass by clasper, and GSI by disk width) was then compared using a Welch's ANOVA for unequal variances, ANOVA, and Kruskal–Wallis test, respectively. Post hoc pairwise comparisons for testes mass by disk width and clasper length residuals were performed using the *glht* function in the *multcomp* package (Hothorn, Bretz, & Westfall, 2008) to account for the unequal variances or unbalanced design, while Wilcox pairwise comparisons were used for GSI residuals to identify significant differences among months. A Bonferroni correction was applied to all pairwise comparisons. To ensure that there was no effect of year on male parameters, we performed the above analyses and tested for significant differences in variance for months where multiyear data contributed to the dataset (i.e., May, June, July, October, and December) using t tests with Welch's correction. We found no significant effect of year (*p* = 1 for all month comparisons), allowing us to pool year by month. All analyses were carried out using R 3.1.0 (R Core Team, 2014).

**Figure 1:**
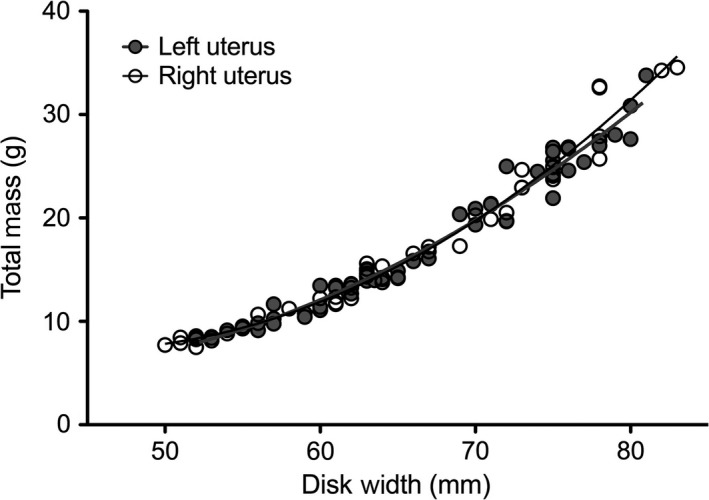
Comparison of pup disk width versus weight for embryos taken from the left (gray circles) and right (open circles) sides. Growth curves from each uterus were found to be nearly identical between the left (gray line) and the right (black line) uterus

In general, multiple paternity studies in elasmobranchs have not taken into account the size or position of embryos in the female reproductive system. However, pooling littermates remove the possibility of identifying differences that could point to evidence of female influence on male fertilization success. Given that some female elasmobranch species are known to have protracted ovulation periods that lead to wide differences in embryo sizes within a litter (Castro, 2000; Kajiura et al., 2000), the opportunity for females to influence sperm access to eggs is a possibility. Therefore, we wanted to explore female ovulation patterns using embryo size as a proxy for timing of ovulation in the round stingray as a potential mechanism by which females could influence multiple paternity. Unfortunately, litter sizes in this study were too small to definitively identify each individual putative father within a litter. Therefore, our aim was to investigate whether there was any merit to exploring the possibility of differences in ovulation among females. We first established that growth was not different between uteri (i.e., females showing uterine investment preferences for one side or the other) by constructing growth curves (i.e., disk width versus weight) by pooling embryos across litters by uterine side (i.e., right or left). As growth was not found to be different (Figure 1), we then described embryo position patterning in uteri among litters, assuming that differences in embryo size were due to fertilization timing differences, to determine whether all females utilize the same patterns. Embryos were assigned rank based on their disk width (ties were assigned the same rank), and litters were categorized as “alternating” or “siding” based on the descending order of embryos’ rank position in the right or left uterus. Alternating litters were designated as those where the descending size order of embryos alternated between the left and the right uterus, whereas siding litters were those where the largest embryos were found on only one side and the smallest on the other. The side of uterus where the largest embryo was found for each designation was noted. Only one litter (PF‐07) did not have position information recorded due to the female aborting her litter prior to euthanasia.

## 3 RESULTS

### 3.1 Multiple paternity

Pups within a litter shared at least one allele per locus with their mother. Simulations in PrDM indicated that the power to detect multiple paternity increased with both litter size and the number of fathers, with two sires for three pups demonstrating the least power and five sires for six pups the greatest (Table 1). Both GERUD 2.0 and COLONY2 revealed that 18 of the 20 litters (90%) were fathered by a minimum of two sires (Table 2). The range in the minimum number of sires provided by GERUD 2.0 was between 1 and 3 with the average number of sires being 2.15 (Table 2). COLONY2 generally estimated a greater number of sires when compared to the minimum number of sires estimated by GERUD 2.0 (13 of 18 litters tested; Table 2). For COLONY2, the putative number of sires ranged between 1 and 5 (Table 2) with the average number of sires being 3.15. Based on COLONY2, the estimated number of putative paternal genotypes contributing to the 20 litters was 27. Of the multiply‐sired litters, 61% (GERUD 2.0) and 56% (COLONY2) were identified as being sired by putative fathers with an equal number of pups per litter (Table 2). When examining only those with fully genotyped litters, the rate of equal sireship contributions increased with seven out of eight litters (~88%) having equal sireship for GERUD 2.0 and five out of eight (~63%) for COLONY2. Each putative father was estimated to have sired an average of 2.08 ± 0.58 and 1.62 ± 0.84 pups per litter for GERUD 2.0 and COLONY2, respectively. Sibship estimates within COLONY2 revealed that the majority of pups were not related (93% of pairwise comparisons), 6% were of a half‐sib nature (*n* = 211/3570 of pairwise comparisons), and 0.9% were full‐sibs (*n* = 37/3570 of pairwise comparisons) (data not shown).

**Table 1:** Results of PrDM simulations for the round stingray that indicate the influence of the effect of sire number, variation in male reproductive success (reproductive skew), and the number of pups within a litter on the estimation of multiple paternity

		Litter size			
# Sires		3	4	5	6
2	(0.50/0.50)	0.667	0.863	0.934	0.967
	(0.67/0.33)	0.595	0.776	0.862	0.909
	(0.75/0.25)	0.500	0.670	0.757	0.817
3	(0.33/0.33/0.33)	0.817	0.956	0.986	0.996
	(0.57/0.29/0.15)	0.723	0.877	0.937	0.965
4	(0.25/0.25/0.25/0.25)		0.980	0.996	0.999
	(0.52/0.27/0.14/0.07)		0.913	0.960	0.978
5	(0.20/0.20/0.20/0.20/0.20)			0.998	1
	(0.50/0.26/0.13/0.07/0.04)			0.966	0.983

Variables used in the simulation include sire number for a given litter (# Sires), potential contribution of various sires to a litter (Reproductive Skew), and the number of pups within a litter (Litter Size).

**Table 2:** Number of sires and paternal skew for the round stingray as estimated by GERUD 2.0 and COLONY2 given the number of embryos genotyped in each litter

Mother	Litter Size	# Sires (G2.0/C2)	Skew (G2.0/C2)
PF04	4	2**/**4	0.50:0.50**/**0.25:0.25:0.25:0.25
PF06	4	2**/**2	0.50:0.50/0.75:0.25
PF07	5	2/2	0.60:0.40/0.80:0.20
PF12	3	2/3	0.66:0.33/0.33:0.33:0.33
PF13	4	1/1	0
PF14	4	2/4	0.50:0.50/0.25:0.25:0.25:0.25
PF15	5	3/5	0.40:0.40:0.20/0.20:0.20:0.20:0.20:0.20
PF17	5	3/5	0.40:0.40:0.20/0.20:0.20:0.20:0.20:0.20
PF18	6	3/5	0.33:0.33:0.33/0.33:0.17:0.17:0.17:0.17
PF20	4	2/4	0.50:0.50/0.25:0.25:0.25:0.25
PF21	5	3/4	0.40:0.40:0.20/0.40:0.20:0.20:0.20
PF28	3	1/2	0/0.66:0.33
PF32	5	2/2	0.60:0.40/0.80:0.20
PF35	4	2/1	0.50:0.50/0
PF37	6	3/4	0.33:0.33:0.33/0.33:0.33:0.17:0.17
PF40	4	2/2	0.50:0.50/0.50:0.50
PF57	4	2/4	0.50:0.50/0.25:0.25:0.25:0.25
PF59	3	2/3	0.66:0.33/0.33:0.33:0.33
PF66	4	2/4	0.50:0.50/0.25:0.25:0.25:0.25
PF68	4	2/2	0.50:0.50/0.75:0.25

Results for the number of sires and reproductive skew from GERUD 2.0 on the left of**/**and from COLONY2 on the right.

### 3.2 Sperm competition

**Figure 2:**
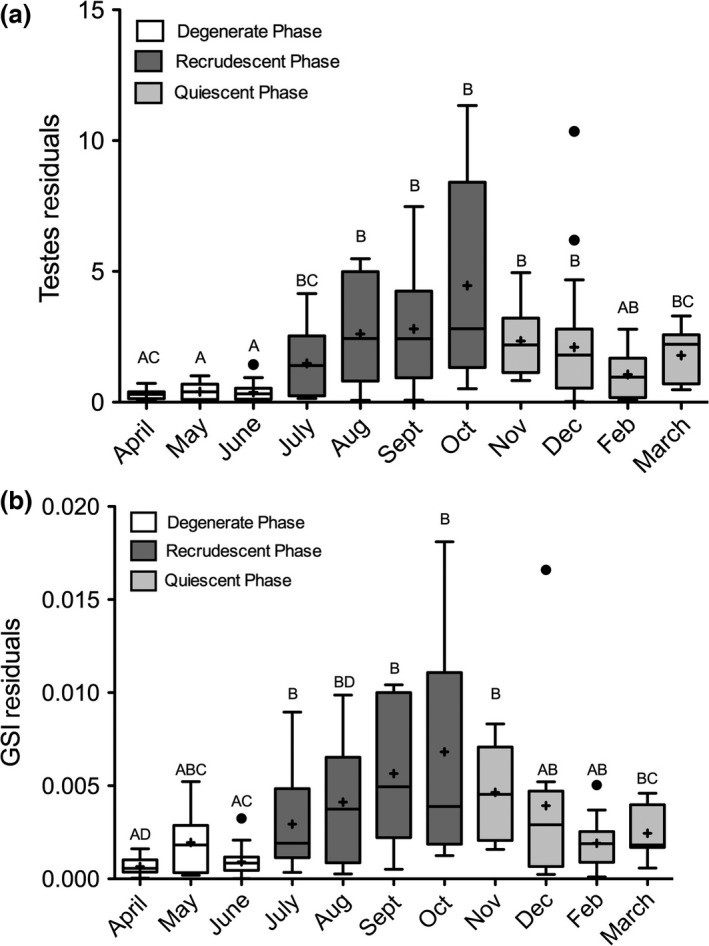
Residuals obtained from linear regressions of testes mass versus disk width (a) and GIS versus disk width (b) exhibit significant differences over time. Boxplots with different letters indicate significant differences (*p* < .05). Dark gray bars denote males in the recrudescent phase (i.e., active spermatogenesis), light gray bars depict males in the degenerate phase (i.e., initiation of cessation of spermatogenesis), and white bars are males in the quiescent phase (i.e., no spermatogenesis)

Male reproductive morphometrics were used to infer the potential degree of sperm competition. We found a significant effect of month on testes mass versus disk width (Welch's ANOVA, *F* = 9.96, numerator *df* = 10, denominator *df* = 48.9, *p* < .0001), testes mass versus clasper length (ANOVA, *F*_10,151_ = 11.282, *p* < .0001), and GSI verses disk width (Kruskal–Wallis chi‐squared = 53.83, *df* = 10, *p* < .0001) in male stingrays. Residual absolute values and variance were lowest during months in the quiescent phase (April–June) and began to rapidly increase (in both value and variance) during the onset of the recrudescent phase (July–October) for all metrics (Figure 2a,b). Values and variances peaked during October, which has been previously noted as the month where male stingray testes are at their highest activity level (Mull et al., 2008). Between April and October, residuals experienced between a 1,300% and 900% increase in absolute value for testes mass versus disk width or clasper length and GSI versus disk width, respectively. After October, residual values and variances began to decline during the degenerate phase, although these months (i.e., Nov to March) were still generally significantly higher than months in the quiescent phase.

### 3.3 Female ovulation patterns

**Figure 3:**
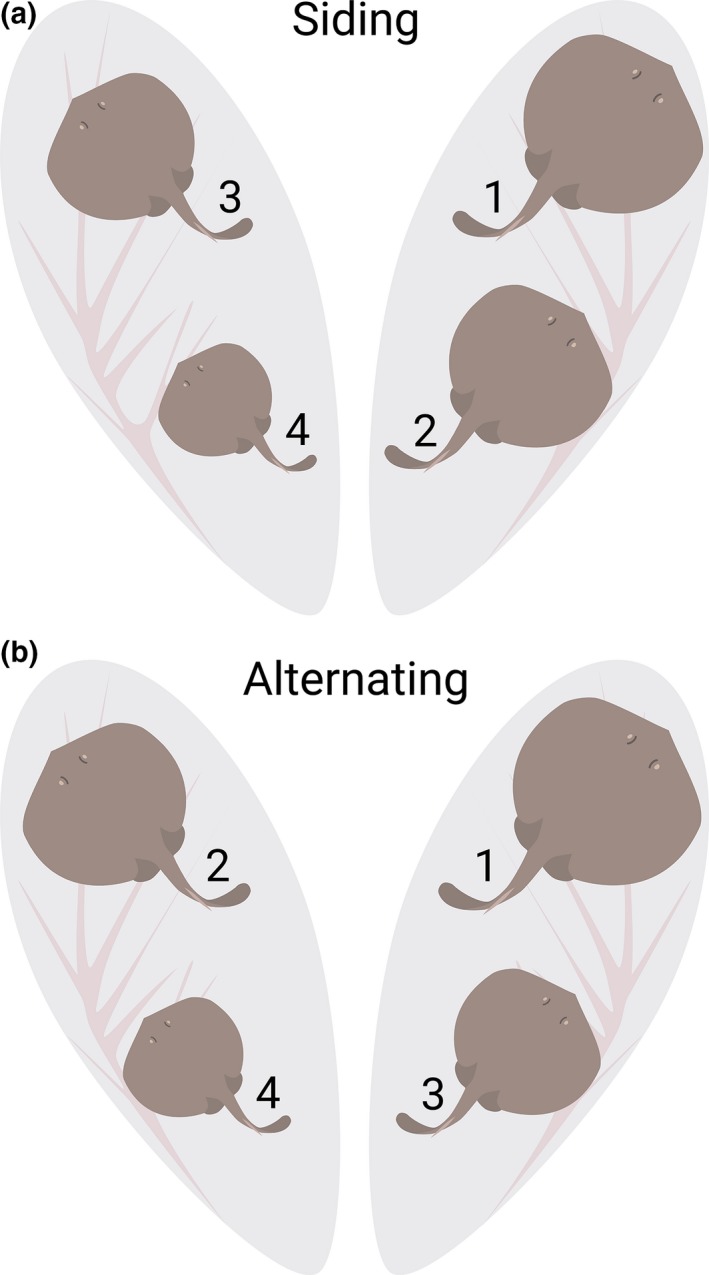
Depiction of the two main categories of female ovulation patterns, “siding” (a) and “alternating” (b), observed in the present study for a litter of four. Upon dissection of pregnant females, the size of embryos was measured (i.e., disk width) and their position in the uterus was noted. Embryos were then ranked based on their size from the largest (1) to the smallest (4) and categorized as “siding” if all of the largest embryos were found in only one uterus or “alternating” if the largest embryos were found on both sides

Despite the high variability in testes mass, suggesting the existence of strong sperm competition among males, sireship success appeared to be generally even among fathers. Given that embryo growth between the left and right uteri was found to be nearly identical among litters (Figure 1), we subsequently assumed that any differences in embryo size were not due to growth differences based on the side of the uterus from which they were found, but differences in timing of fertilization. Therefore, examining embryo position differences based on size within litters gave us the opportunity to make inferences on female ovulation patterns. Of the 19 litters examined, we found that approximately 58% exhibited a siding pattern and 42% exhibited an alternating pattern (Figure 3a and b, respectively); however, within each of these two main categories, embryo size positioning patterns were not exactly the same for litters with the same number of embryos (Figure S1). The most commonly occurring pattern (*n* = 3 litters) was where the largest and second largest embryos were found in the left uterus and the next two smallest embryos were found in the right uterus (Figure 3a). Other than this particular pattern, every other litter demonstrated a slightly different variation of one of the two main pattern categories. For both siding and alternating litters, the largest embryo of a litter was more often found in the left uterus than in the right where litters demonstrating siding had the largest embryo on the left in 73% of observations, whereas for litters with an alternating pattern, the largest embryo was more evenly split between the left (43%), right (28.5%), and the left and right having embryos of the same exact size (28.5%). The size difference between the largest and smallest embryo within a litter (left and right uterus combined) was similar for both siding (2‐7 mm, median 4 mm) and alternating litters (2–6 mm, median 3.75 mm). Using COLONY2 paternal assignments, full siblings in different uteri (i.e., right or left) differed 0–6 mm in disk width (median: 2 mm); however, these differences were not significantly greater than full siblings in the same uterus (*p* = .10), although the range in size difference was less (0–3 mm).

**Figure 4:**
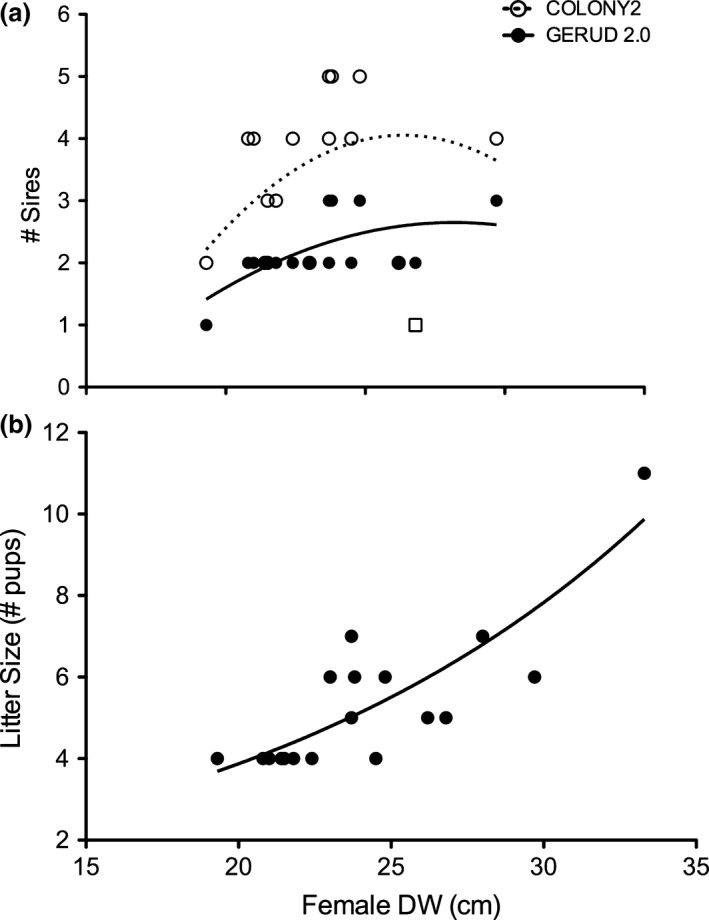
Relationship of female disk width (DW) size with respect to number of sires (a) and litter size( )(b). (a) Sireships generated from GERUD 2.0 (closed circles, solid line) and COLONY2 (open circles, dashed line) are for fully sequenced litters and litters where only one pup was unable to be sequenced (*n* = 17 out of 20 litters). Note, the parabolic relationship generated for the COLONY2 dataset holds only when the PF35 litter is excluded (open square). (b) Female fecundity generally increases with size in an exponential fashion

The number of putative sires per litter was plotted against female size to investigate any relationship between female mating experience (assuming that larger, older females will have undergone more reproductive cycles when compared to smaller, younger females) and number of successful mating attempts (Figure 4a). For both GERUD 2.0 and COLONY2, a parabolic relationship appeared to exist with number of sires increasing with size (i.e., age) and plateauing or decreasing, respectively. When only females with litters larger than three pups were examined, no relationship was found between female size and number of putative sires. However, female fecundity strongly increased with size (Figure 4b). Furthermore, when females were separated by “even sireship” and “skewed sireship” and their sizes were compared, there was no significant difference (GERUD 2.0: Student's t test t_15_ = 0.46, *p* = .65; COLONY2: Welch's t_7_ = 0.13, *p* = .24). Initially, we predicted that smaller females would have less skewed litters as a result of their inability to fend off males (resulting in multiple matings) and older, larger females would have more skewed litters due to their ability to be more “choosy” and select higher quality males. However, we did not find any relationship between female size and the skewness of the litter suggesting that males are not necessarily better at coercing younger females to mate than older females.

## 4 DISCUSSION

Our study is one of the first to suggest that both intra‐ and intersexual selection play a role in the occurrence of multiple paternity in an elasmobranch species. Multiple paternity was found to occur at a high rate, and there was a high incidence of litters being composed of mostly half siblings (Table 2). Despite indications that reproductive success (i.e., high testes variability) is unequal among males, a situation that we hypothesized would lead to greater skew in multiply‐sired litters, we unexpectedly found sireship to be relatively even among litters. However, the power to definitively assess reproductive skew based on our data is limited by litter size and we are merely indicating the detection of a potential pattern that will require further investigation. Our observations from the female perspective suggest that female round stingrays may play a role in influencing sperm access to eggs through differential ovulation patterns.

In previous studies, the high rate of multiple paternity in elasmobranchs has been attributed to convenience polyandry (DiBattista et al., 2008; Griffiths et al., 2011) or lack of female control over mate choice and/or frequency (Chapman et al., 2013). Therefore, multiple paternity may be high when populations are large with a high mate encounter rate (Chabot & Haggin, 2014; Daly‐Engel, Grubbs, Bowen, & Toonen, 2007; Kokko & Rankin, 2006; Soucy & Travis, 2003). Indeed, the mobility and large population of round stingrays may make it difficult for any one male to dominate or exclude other males, and our data demonstrate that there is great variation in male reproductive potential (i.e., high variation in testes mass), suggesting that males perceive their mating opportunities to be reduced (Arnqvist, 1992) and competition to be high. In many taxa, increased sperm competition, or the perception of high competition, is a strong driver of testes mass and variability (Firman & Simmons, 2008; Møller, 1998; Simmons, 2001). Both testes mass and GSI variability were influenced by month of the year and dramatically increased during months within the recrudescent phase in round stingrays. While gonad size clearly scales with male body size, the higher absolute residual values and wide variance of these values during the recrudescent period suggest that males perceive their reproductive success to be unequal compared to other conspecifics. Although previous studies have not quantified the relationship between sperm production and testes mass in round stingrays, assuming absolute testes mass to be positively related to sperm production (Gage, Stockley, & Parker, 1995), we suggest that sperm competition may be strong in round stingrays and that reproductive success in males could be highly skewed toward larger (i.e., older) males with more mating experience and increased sperm production (Harcourt et al., 1981; Parker et al., 1997). It is also important to consider that male elasmobranchs have two intromittent organs (claspers) and may vary in whether they insert either one or both into a female during a mating event (Pratt & Carrier, 2001). Therefore, it is possible that males may not have access to an entire litter, depending on their ability to use one or both claspers. Despite indications of strong sperm competition, reproductive success was found to be fairly even and contradicted expectations that competition, as inferred from testes mass variation, would have led to skewed reproductive success (i.e., one male dominating sireship), suggesting that other potential mechanisms may be utilized.

The high incidence of multiple paternity may be related to conflict between males and females and the strategies that each employs to maximize fitness. Mating with multiple males has been attributed to the generally aggressive nature of elasmobranch males toward females during elasmobranch courtship (Chapman et al., 2013; DiBattista et al., 2008; Feldheim et al., 2004; Kajiura et al., 2000; Nordell, 1994; Pratt & Carrier, 2001). However, this implies that females play a passive role and have no influence in mate selection or choice, despite the potential of physiological or behavioral mechanisms to give females the opportunity to exert sireship choice by limiting or influencing the window of opportunity in which sperm can fertilize eggs. The assumption that females play no role in sperm selection, by any means, seems questionable considering the high investment females put into producing well‐developed young as well as suggestions that females, in general, have the capacity to influence sperm selection (Birkhead, 1998). For instance, in a population of fowl (*Gallus gallus domesticus*) where females are coerced into mating with subordinate males, females were found to differentially expel sperm from males based on their social rankings (Pizzari & Birkhead, 2000). Alternatively, studies in mammals have implicated sperm–female reproductive tract interactions as playing a role in sperm selection by females (Holt & Fazeli, 2010). Given the various opportunities between mating and fertilization afforded to females to exert mate choice as seen in other vertebrate taxa, it would be surprising if elasmobranch females have no mechanism to control male fertilization success.

In the present study, we observed a variety of embryo positioning patterns, which we assume are related to the timing of ovulation of eggs, and we argue could represent a potential mechanism by which female round stingrays control male access to litters. From the wide range in embryo size, it seems unlikely that females ovulate all of their eggs at once and then proceed to mate, but rather sequentially ovulate their eggs periodically with mating occurring throughout this process. If eggs were all ovulated in close succession, we would expect to see higher rates of skewed paternity (i.e., one male fertilizing a majority of a litter) than the more even paternity we observed as well as reduced variance in littermate disk width. Female manipulation of sperm access to eggs by differential ovulation patterns could represent a level of control to influence the ability of certain males to fertilize their eggs, a hypothesis previously proposed by Heist et al. (2011). The lack of homogeneity in apparent ovulation patterns highlights the plasticity of this physiological trait in female round stingrays and suggests this could be a potential consideration for other elasmobranch species.

Generally, female receptiveness occurs over a window of time, and females of some species have been documented to have prolonged periods of ovulation (Castro, 2000; Kajiura et al., 2000). This would be expected to increase the opportunity for multiple males to sire any given litter (Heist et al., 2011). While unconfirmed in round stingrays (Babel, 1967), females of some species have been demonstrated to store sperm (Pratt, 1993), which would add an additional opportunity for females to exert mate choice postcopulation and should be further investigated. Variation in reproductive physiology among female elasmobranchs suggests that other mechanisms influencing sperm access to eggs may influence multiple paternity, rather than solely male‐related factors (i.e., male aggressiveness, encounter rates). Future work in other elasmobranch species should investigate the relationship between ovulation patterns and multiple paternity in species where the ability to distinguish putative sires within a litter has greater confidence (i.e., in species with large litter sizes) than the present study species.

Round stingrays appear to exhibit a combination of intra‐ and intersexual selection that may contribute to the observed high occurrence of multiple paternity in this species and suggests that elasmobranch mating systems may be more complex than previously acknowledged. The high incidence of multiple paternity, large variation in male testes mass, and potential for differential ovulation patterns leads us to propose a working hypothesis for the mating system of this species where conflict is strong both among males and between males and females, with both sexes working to influence paternal litter sireship. While strong sperm competition generally leads to highly skewed reproductive success, this may be overridden by variation in female ovulatory patterns as a mechanism leading to a system where reproductive evenness is maintained and prevents individual males from siring the majority of a litter. Taken together, our results indicate that future studies investigating mechanisms of multiple paternity should delve deeper into the role that both males and females play in promoting this system. Considering the wide variety of both niches and reproductive strategies (i.e., egg laying to placental) that elasmobranchs utilize, and that our study represents only one combination of these factors, the possibility to examine sexual selection and reproductive success across members of this taxon opens up an entire field of study that is expected to enhance our understanding of the factors that influence mating systems within the animal world.

## CONFLICT OF INTEREST

The authors declare no conflict of interest.
