# Supplementary figures and images for: Who's My Daddy? Considerations for the influence of sexual selection on multiple paternity in elasmobranch mating systems
Source: Ecol Evol. 2017 Jun 15;7(15):5603–12. doi: 10.1002/ece3.3086 (PMC5551082; doi:10.1002/ece3.3086)

# Siding

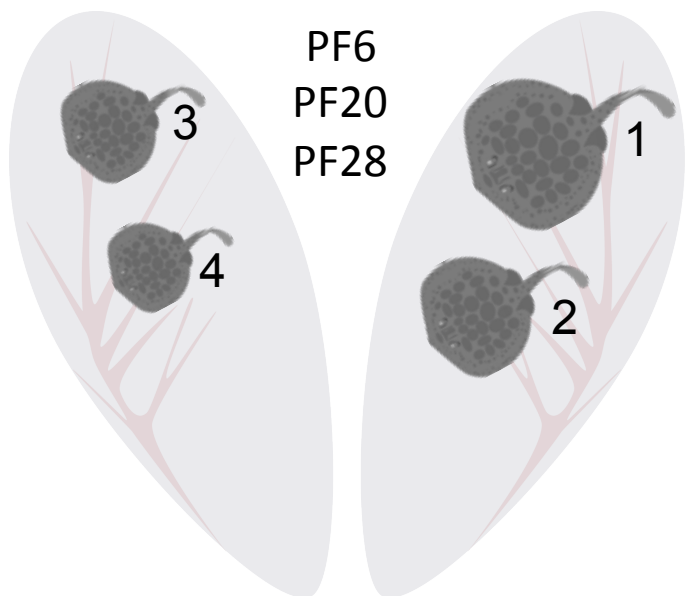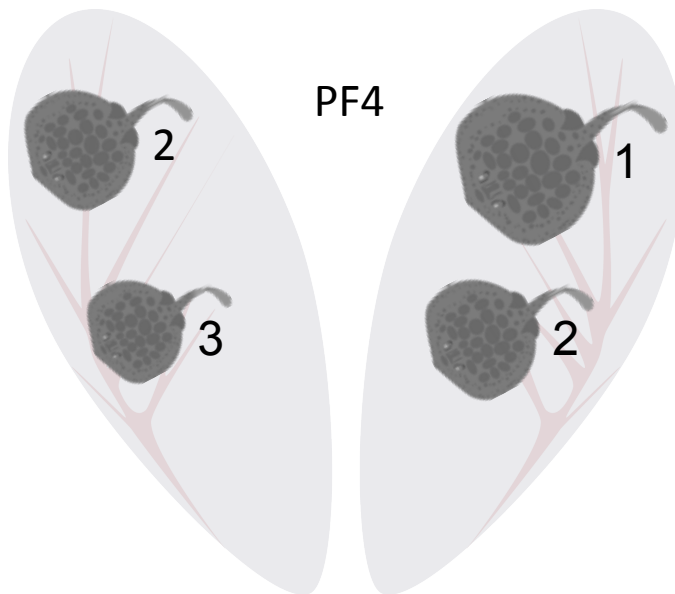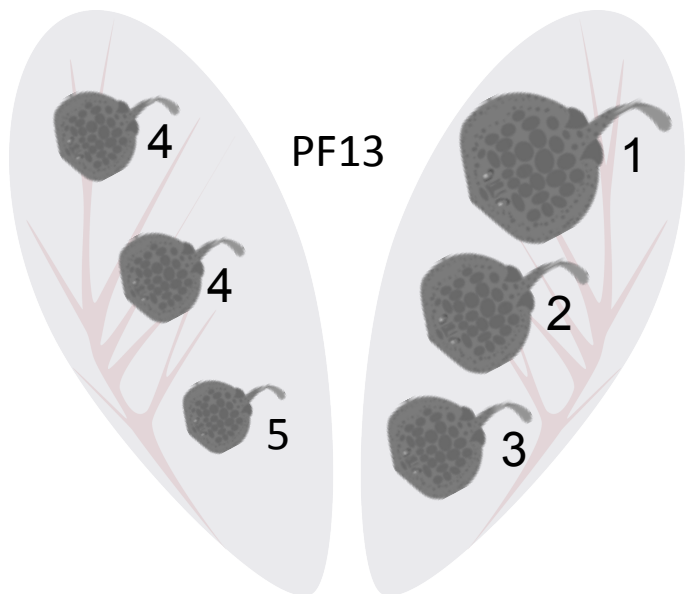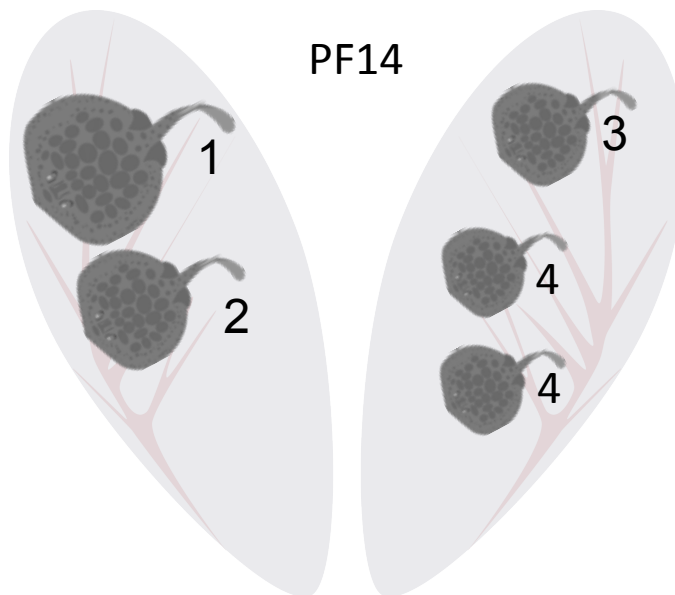

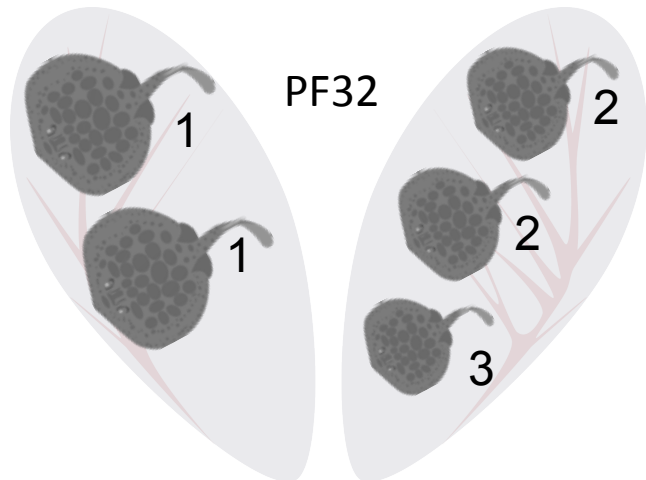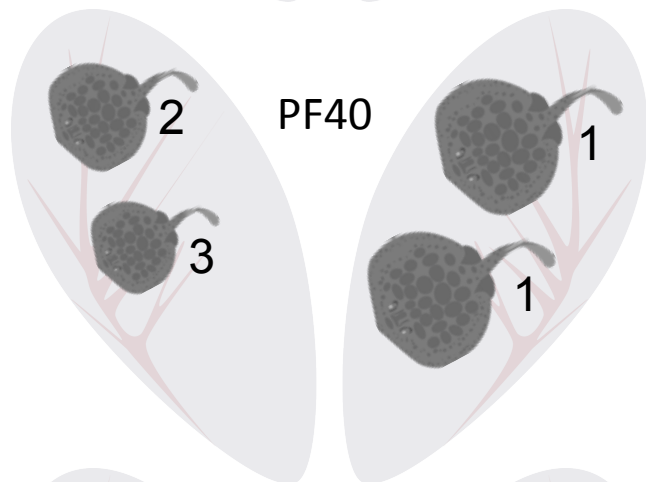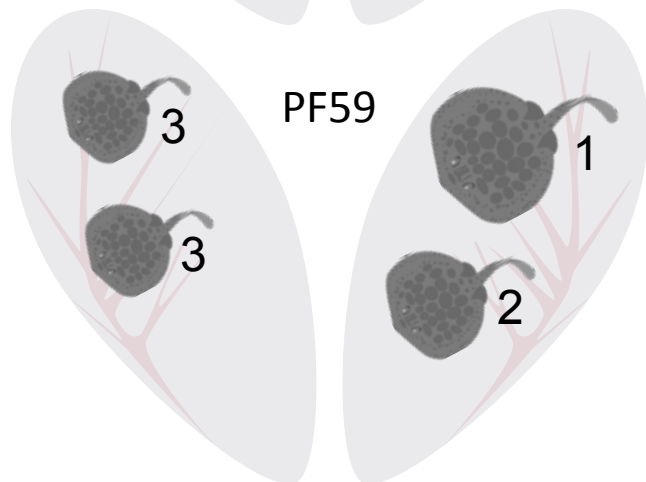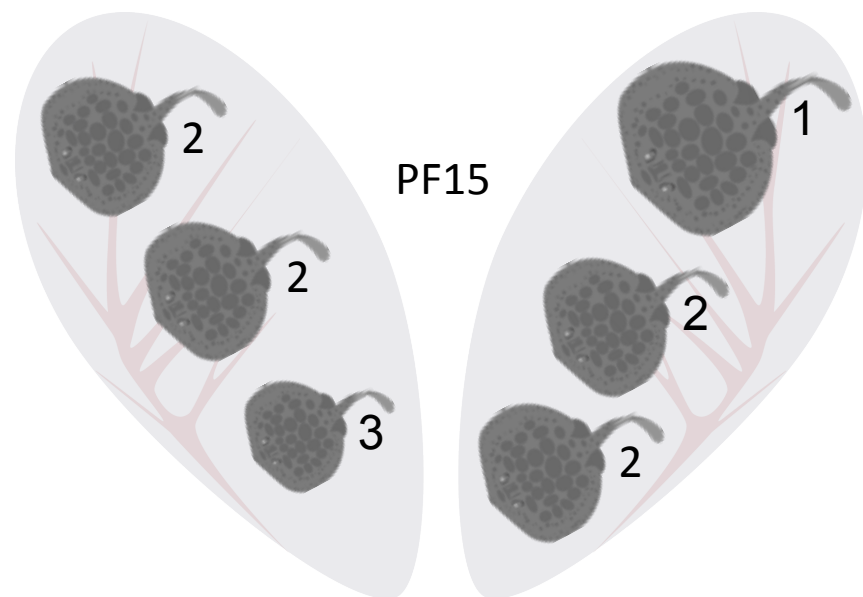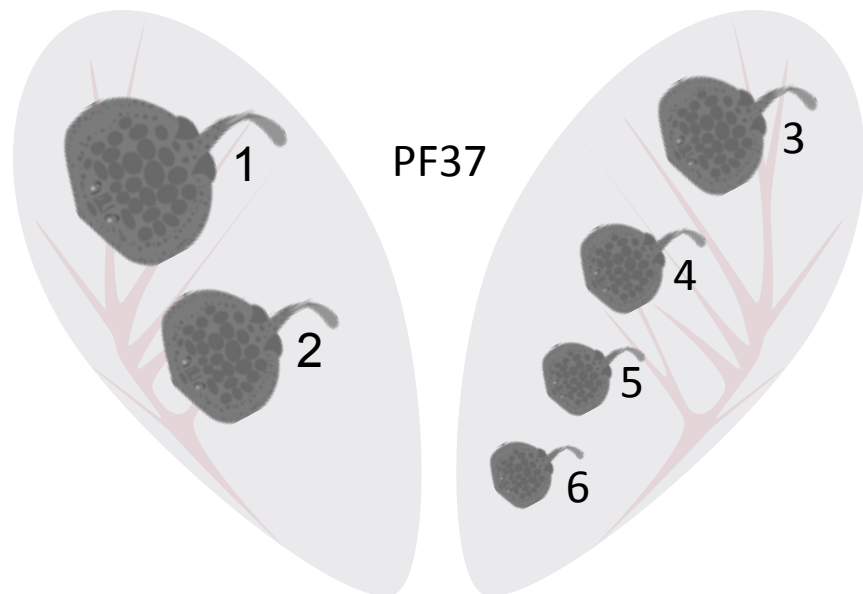

# Alternating

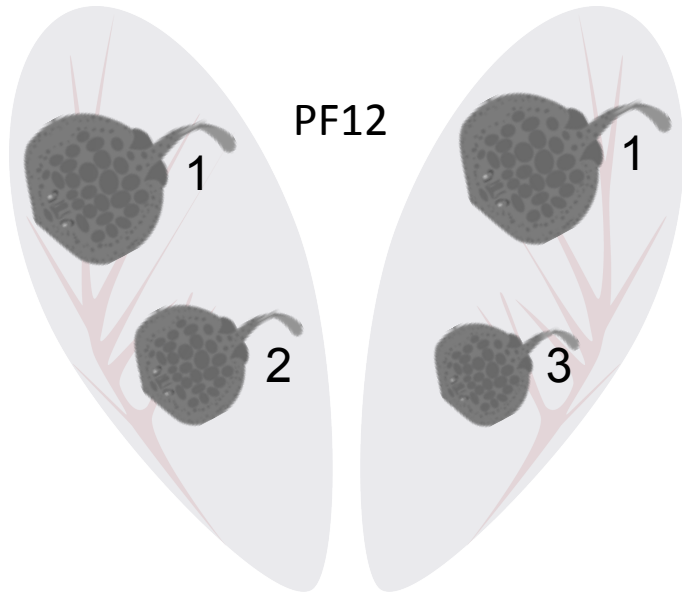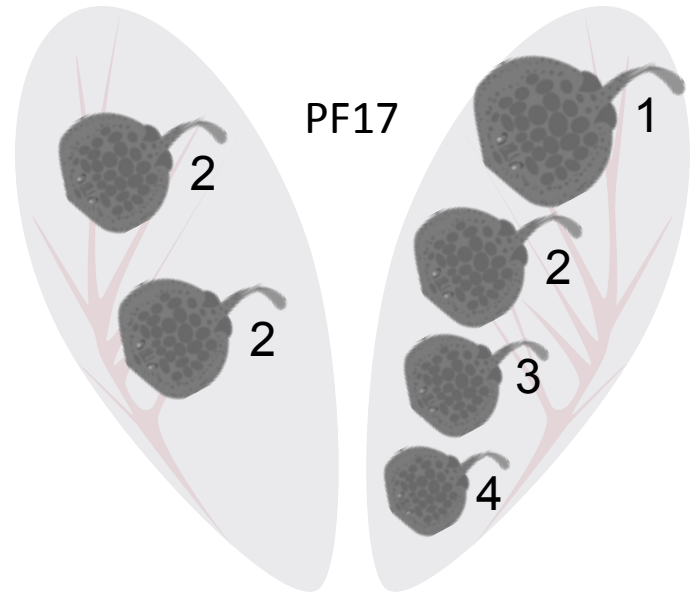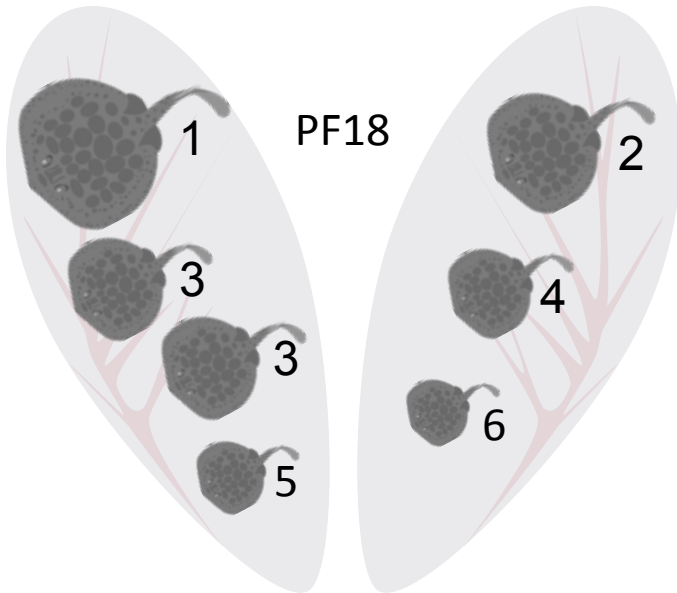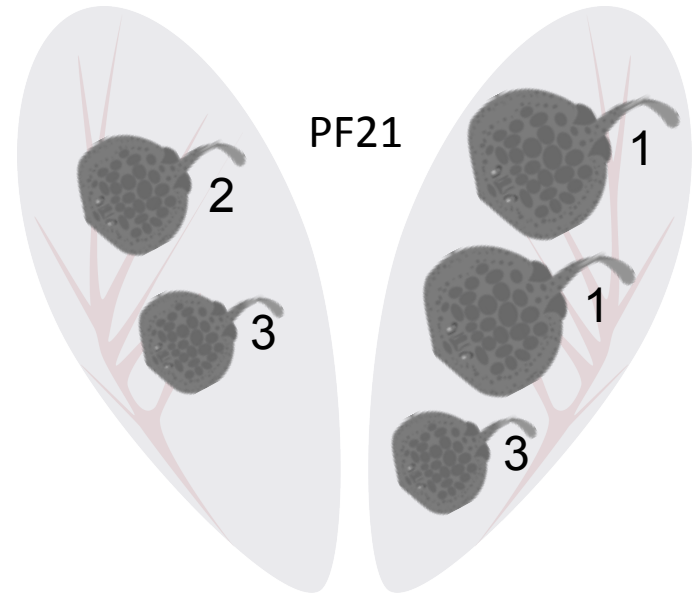

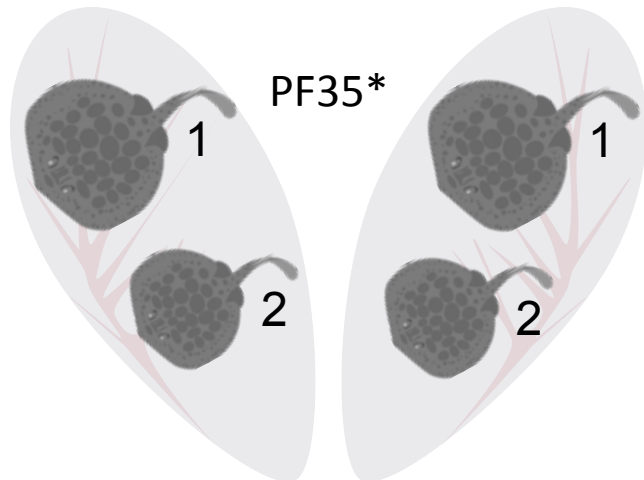

PF35\*

\*Note: largest pup uterus designation is missing

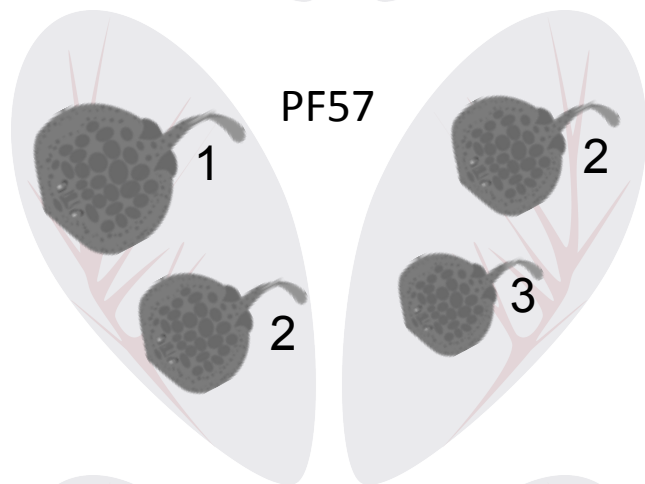

PF57

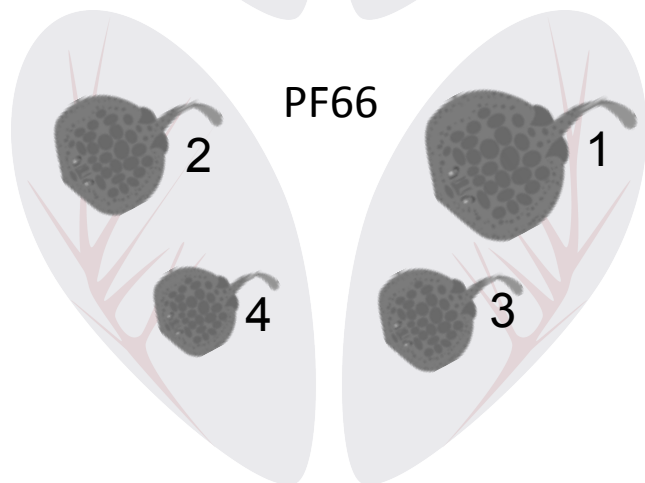

PF66

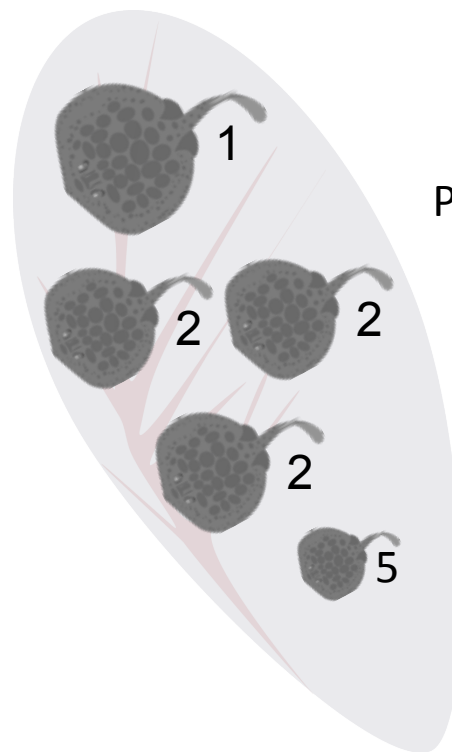

PF68

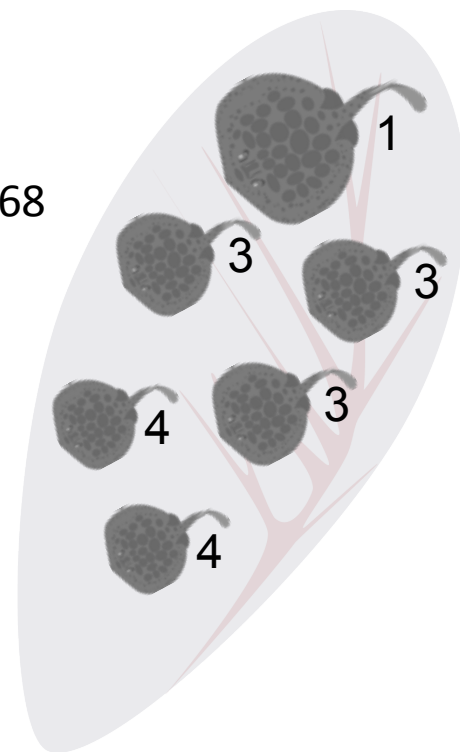

Supplement: Supplementary file 1 [file ECE3-7-5603-s001.pdf]
